# Modified Glasgow prognostic score can predict survival of muscle invasive bladder cancer patients after radiotherapy

**DOI:** 10.1093/jrr/rraa039

**Published:** 2020-06-22

**Authors:** Koyo Kikuchi, Ryuji Nakamura, Takafumi Segawa, Hirobumi Oikawa, Hisanori Ariga

**Affiliations:** Department of Radiation Oncology, Iwate Medical University School of Medicine, 2-1-1 Idaidori, Yahaba-cho, Shiwa-gun, Iwate 028-3695, Japan

**Keywords:** Bladder cancer, External beam radiotherapy, Bladder preservation therapy, modified Glasgow prognostic score

## Abstract

In patients with various cancers, modified Glasgow prognostic score (mGPS) before treatment has predicted prognoses after antitumor therapy. This study aimed to assess whether pretreatment mGPS also has predictive value in patients with muscle-invasive bladder cancer (MIBC) after radiotherapy. A retrospective review accumulated 98 consecutive MIBC patients treated with definitive 3D-conformal radiotherapy from January 2011 to December 2016 in a single center. It included cT2-4bN0-3M0 patients with a median age of 79 years (range: 49 to 95 years). Radiotherapy was delivered at 60–66 Gy for bladder cancer. Patients were categorized in terms of their pretreatment serum albumin and C-reactive protein (CRP) values as mGPS_0, mGPS_1, and mGPS_2. Among them, cumulative overall survival (OS) rates were compared by Kaplan–Meier plots with log-rank tests. The number of patients with mGPS_0, mGPS_1, and mGPS_2 were 40, 40, and 18, respectively. The median follow-up time for all patients was 19 months (range: 2–73 months). The 2-year OS rate for all patients was 75.7%. The 2-year OS rates for mGPS_0, mGPS_1, and mGPS_2 were 85.1%, 71.3%, and 60.9%, respectively. Kaplan–Meier curves revealed a significantly higher cumulative OS rate for mGPS_0 compared with mGPS_1 and mGPS_2 (*P* = 0.003). Using multivariate Cox regression analysis, mGPS_0 and good performance status were associated with favorable OS rates, of which mGPS_0 was more significant (Hazard ratio 2.74, 95% CI 1.30–5.57, *P* = 0.008). Modified Glasgow prognostic score may be a novel biomarker that can predict survival in patients with MIBC after radiotherapy.

## Introduction

Bladder cancer is the tenth most common cancer in the world, with 550,000 new cases diagnosed in 2018 [1]. Bladder cancer typically occurs in elderly patients and it is becoming a major societal issue in Japan, which has already become an aging society [2]. At the time of diagnosis, approximately 25% of bladder cancer patients present with muscle-invasive bladder cancer (MIBC). Although cisplatin-based neoadjuvant chemotherapy followed by radical cystectomy has been the standard approach to the treatment of MIBC, a recent meta-analysis revealed no differences in overall survival (OS) rates, disease-specific survival rates, or progression-free survival rates between radical cystectomy and transurethral resection of the bladder tumor (TUR-Bt) followed by chemoradiotherapy. Therefore, radiotherapy has the possibility of being alternative option for MIBC that is less invasive and bladder preserving approach [3].

The Glasgow prognostic score (GPS) is a biomarker indicating the degree of systemic inflammation determined by a value derived from serum albumin and C-reactive protein (CRP) levels. It was initially reported by McMillan et al. [4] in 2003 using non-small cell lung cancer patients. They revealed that its predictive value was superior to other factors such as patient performance status or tumor clinical stage. Miki et al. [5, 6] validated GPS for Japanese patients by considering differences in assay kits and physique. They proposed a modified GPS (mGPS) with a cutoff value for CRP of 0.5 mg/dL. Some researchers reported that GPS or mGPS can predict survival rates in several cancers, such as lung cancer, gastrointestinal cancer, hepatobiliary-pancreatic cancer, and bladder cancer after surgery or chemotherapy [4, 6–12]. As a post-radiotherapeutic prognostic factor, Kishi et al. [13] revealed that mGPS was a significant predictor of clinical outcome in stereotactic body radiation therapy for non-small cell lung cancer. However, the predictive value of mGPS in radiotherapy for bladder cancer has not yet been established. Therefore, this study aimed to evaluate the usefulness of mGPS as a prognostic factor in patients with MIBC treated with radiotherapy.

**Table 1:** Patients’ characteristics

characteristics		mGPS_0 (n = 40)	mGPS_1 (n = 40)	mGPS_2 (n = 18)	Total (n = 98)
Median age, years (range)		78 (51–95)	79 (49–87)	82 (66–87)	79 (49–95)
Sex	Male/Female	30/10	32/8	15/3	77/21
Pathology	Urothelial ca.	37	38	16	91
	Squamous ca.	1		2	3
	Adeno ca.	1	1		2
	Undifferentiated ca.	1			1
	Unknown		1		1
ECOG-PS	0/1/2/3	17/17/6/0	15/15/8/2	6/5/6/1	38/37/20/3
Stage	II/III/IV	13/23/4	14/21/5	5/10/3	32/54/12
Dose (Gy)	60/61.2–65/66	14/3/23	17/4/19	9/2/7	40/9/49
Concurrent chemoradiotherapy		6 (15%)	8 (20%)	2 (11%)	16 (16%)
Median follow up, months					19 (2–73)

## Materials and Methods

This was a retrospective, single-center study at Iwate Medical University Hospital and was approved by the Institutional Review Board on December 2018 (MH2018–572). A total of 115 patients with bladder cancer were treated with definitive radiotherapy at our institution between January 2011 and December 2016. We analyzed patients who had been evaluated for serum albumin and CRP before radiotherapy for MIBC in clinical stage II–IV without distant metastases according to the TNM classification of malignant tumors (7th edition) prescribed by the Union for International Cancer Control.

### Patients

Ninety-eight patients (77 men and 21 women) diagnosed with cT2-4aN0-3M0 were enrolled in this study. Patient characteristics are shown in Table 1. The median age was 79 years old at the time of radiotherapy and 90 patients (92%) were considered elderly (≥65 years). In addition, 60 patients (61%) had some disabilities as graded by Eastern Cooperative Oncology Group Performance Status (ECOG-PS). Approximately half of the patients (51%) had one or more comorbidities, including cardiovascular disease (n = 27), diabetes mellitus (n = 18), chronic cerebral infarction/hemorrhage (n = 27) or chronic kidney disease (n = 11). Thirty patients (31%) had a previous or concurrent history of other primary malignancies. Five of them had three or more primary malignancies including bladder cancer. The details are as follows: gastric cancer (n = 7), prostate cancer (n = 6), ureteral cancer (n = 5), colon cancer (n = 3), hepatocellular carcinoma (n = 2), lung cancer (n = 2), breast cancer (n = 2), esophageal cancer (n = 2), malignant lymphoma (n = 2), renal cell carcinoma (n = 1), endometrial carcinoma (n = 1), adenoid cystic carcinoma of mandible (n = 1), hypopharyngeal cancer (n = 1), and gallbladder cancer (n = 1). Eleven patients had metastasis in pelvic lymph nodes and 32 patients exhibited hydronephrosis. In 27 of the patients, TUR-Bt was performed at a median of 1 time (range 1–4). In six patients, intravesical bacillus Calmette–Guérin immunotherapy was administered following TUR-Bt. These patients were treated with definitive 3D-conformal radiotherapy (3D-CRT) instead of cystectomy because of their advanced age, medical comorbidities, refusal of surgery, or unwillingness to undergo surgery.

**Table 2:** 

	Univariate analysis (log-rank test)	Multivariate analysis (Cox regression analysis)	
Factor	*P* value	HR (95% CI)	*P* value
Age, years ≥80 vs < 80	0.240		
Sex Male vs Female	0.610		
ECOG-PS 1,0 vs 2,3	0.003	0.39 (0.18–0.83)	0.014
Clinical stage II vs III,IV	0.937		
TUR-Bt followed by radiotherapy yes vs no	0.884		
Concurrent chemoradiotherapy yes vs no	0.647		
Irradiation field Whole bladder vs Small pelvis	0.994		
Prescribed dose (Gy) ≥64 vs < 64	0.047	0.59 (0.30–1.17)	0.132
mGPS 0 vs 1,2	0.003	0.37 (0.17–0.77)	0.008

*Abbreviations:* mGPS = modified Glasgow prognostic score, ECOG-PS = Eastern Cooperative Oncology Group Performance Status, TUR-Bt = Transurethral resection of bladder tumor.

### Treatment

Computed tomography images of the pelvis were obtained from patients in the supine position and were imported into a radiation treatment planning system (Eclipse ver. 11.0: Varian, Palo Alto, CA.). A 3D-CRT was performed with 10-MV photons using a rectangular 4-field technique with a linear accelerator (Clinac iX or Clinac 2100: Varian, Palo Alto, CA.) for 60–66 Gy at 1.8–2.0 Gy, once daily fractions over 6–7 weeks. We initiated 3D-CRT with 40 to 46 Gy toward the small pelvis (n = 26) for patients with clinically positive or suspected lymph node metastases, or whole bladder (n = 70), otherwise for empty bladder. Then, image-guided radiotherapy was increased to 66 Gy for bladder cancer and principally performed under full bladder by a consistent preparation. For patients with multiple bladder lesions and/or urinary incontinence, whole bladder irradiation was performed up to 60 Gy. Two patients, one with bladder adenocarcinoma and the other very elderly (90 years old), received 66 Gy of image-guided radiotherapy for bladder cancer without prophylactic irradiation.

Sixteen patients were treated with concurrent chemoradiotherapy including gemcitabine plus cisplatin (gemcitabine: 1000 mg/m^2^ on days 1, 8, 15; cisplatin: 70 mg/m^2^ on day 2) (n = 10), gemcitabine plus carboplatin [gemcitabine: 1000 mg/m^2^ on days 1, 8, 15. carboplatin: area under the curve (AUC) of 5 on day 2] (n = 3), monthly cisplatin (70 mg/m^2^) (n = 1), weekly carboplatin plus paclitaxel (carboplatin: AUC of 2; paclitaxel: 40 mg/m^2^) (n = 1), and tegafur/uracil (tegafur: 300 mg/m^2^/day) (n = 1). All patients periodically visited the clinic for outcome assessment and adverse effect management. The Common Terminology Criteria for Adverse Events, version 4.0, grading system was used to evaluate the severity of genitourinary or gastrointestinal toxicities. Toxicities that occurred during or within the first 3 months after radiotherapy were classified as acute adverse events, and those that occurred ≥3 months after radiotherapy were categorized as late adverse events. The initial effects of the radiotherapy on the bladder tumors were classified according to the Response Evaluation Criteria in Solid Tumors, version 1.1.

### Modified Glasgow Prognostic Score

We assessed the laboratory data of patients obtained before radiotherapy. The median time from the date of serum analysis to the start of radiotherapy was 6 days (0–66 days). The median value of serum albumin and CRP before radiotherapy were 3.6 g/dL (range, 2.1–5.1 g/dL) and 0.3 mg/dL (range, 0.0–9.9 mg/dL), respectively. Based on this data, patients were divided into three subgroups: mGPS_0, mGPS_1, and mGPS_2, which were defined by the composition values CRP < 0.5 mg/dL and albumin ≥3.5 g/dL, CRP ≥0.5 mg/dL or albumin < 3.5 g/dL, and CRP ≥0.5 mg/dL and albumin < 3.5 g/dL, respectively.

### Statistical Analysis

OS was defined as the time from the start of radiotherapy to death from any cause. OS rates were compared using Kaplan–Meier estimates with log-rank test verification as univariate analyses for age (≥80 vs < 80 years), sex (male vs female), ECOG-PS (0,1 vs. 2,3), clinical stage (stage II vs III-IV), with or without TUR-Bt followed by radiotherapy, with or without concurrent chemotherapy, irradiation field (whole bladder vs small pelvis), prescribed dose (≥64 vs < 64 Gy), and mGPS (mGPS_0 vs mGPS_1,2). Multivariate Cox proportional hazards regression analysis was used to estimate hazard ratios (HR) for all factors significant from the univariate analysis. A *p*-value less than 0.05 was considered statistically significant. All analyses were conducted using SPSS software version 20.0 (Chicago, IL, USA, August 2011).

## Results

All patients completed radiotherapy up to the prescribed doses with no delay beyond a week. The initial tumor responses were evaluated as a complete response (n = 39), partial response (n = 38), stable disease (n = 7), progressive disease (n = 1), or unknown due to unavailable posttreatment images (n = 13). There was no significant difference in response rates between patients prescribed the higher dose (≥64 Gy) or the lower dose (<64 Gy) of radiation (91% vs 90%, respectively). Bladder cancer recurred at a mean disease-free interval of 15 months after therapy as bladder alone (n = 34), distant metastasis alone (n = 12), or bladder with distant metastasis (n = 11). The sites of bladder cancer recurrence were usually identical to the primary sites. Forty-one patients suffered acute urinary symptoms including pollakisuria with urgency or retention, or micturition pain. Eleven patients developed hemorrhagic cystitis as a late toxicity. During the follow-up period, 36 patients died. Twenty-five patients died due to bladder cancer, four patients died of cardiovascular events, four patients died of other cancers, one patient died of drug-induced pneumonia, one patient died of hemoptysis, and the remaining patient died of an unknown cause.

**Fig. 1.**
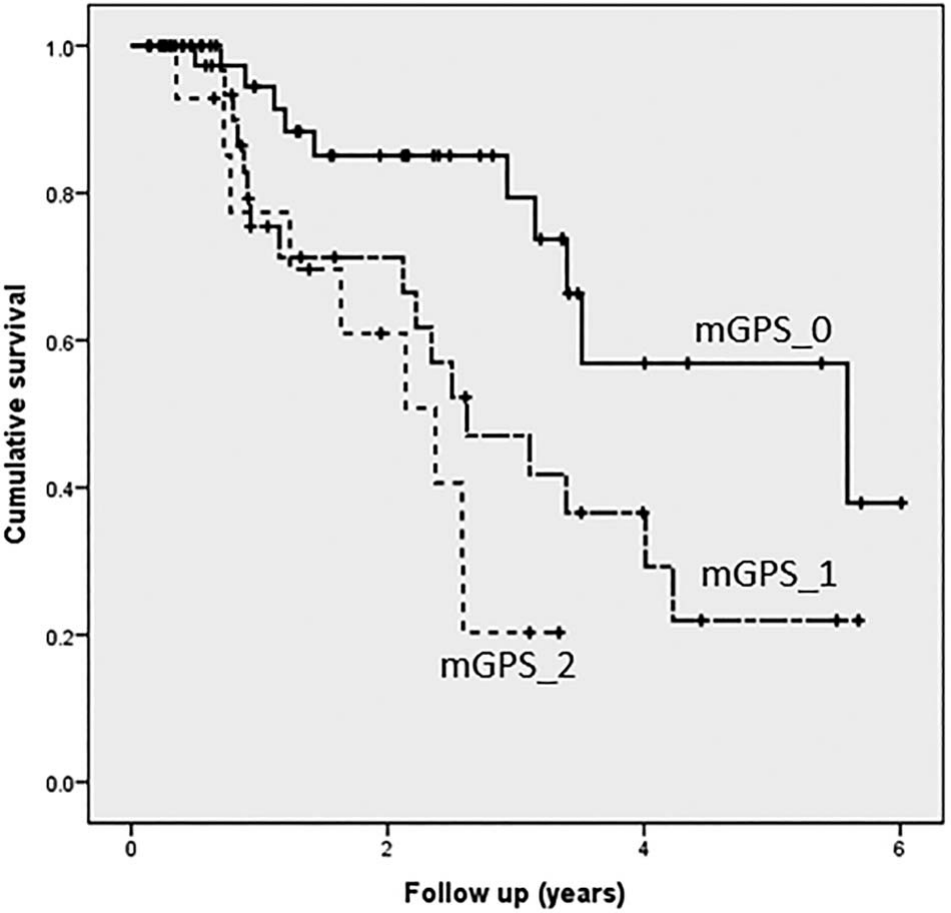
The 2-year OS rate for all patients, mGPS_0, mGPS_1, and mGPS_2 were 75.7%, 85.1%, 71.3%, and 60.9%, respectively.

The number of patients with mGPS_0, mGPS_1, and mGPS_2 were 40, 40, and 18, respectively (Table 2). The median follow up for all patients who were alive at the time of the last follow up was 19 months (range: 2–73 months). The 2-year OS rates for all patients at mGPS_0, mGPS_1, and mGPS_2 were 75.7%, 85.1%, 71.3%, and 60.9%, respectively (Fig. 1).

**Fig. 2.**
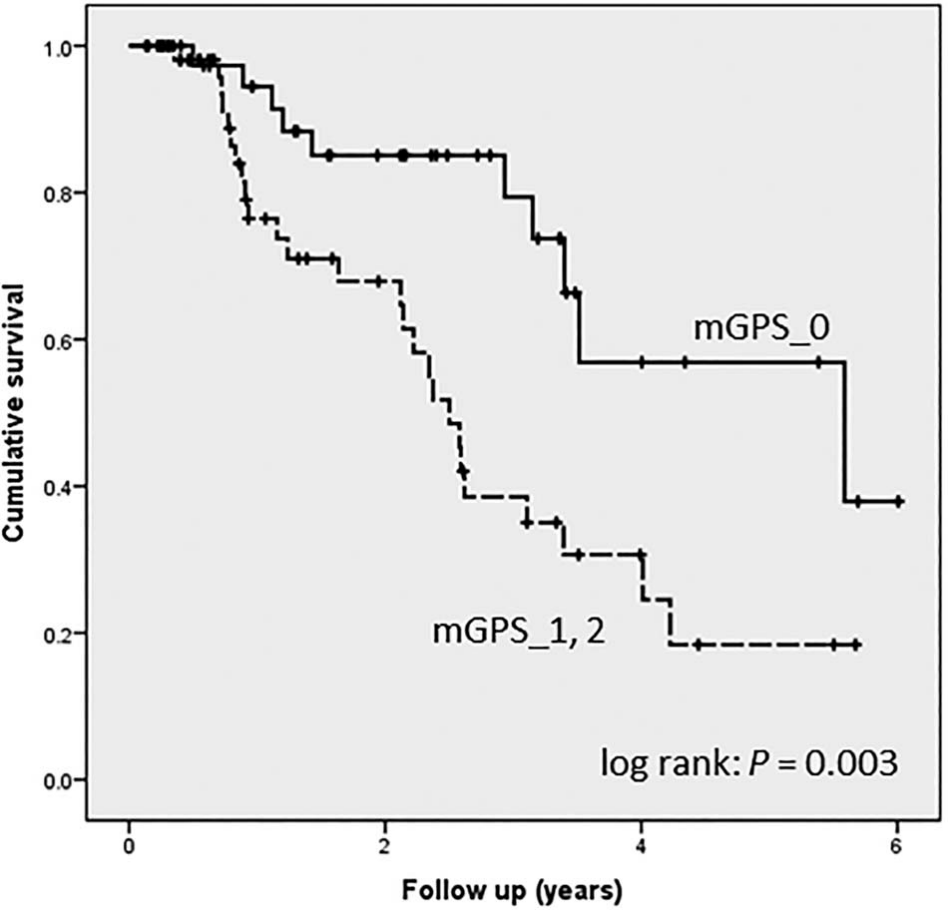
Kaplan–Meier curves revealed a significantly higher cumulative survival rate for mGPS_0 compared with mGPS_1 and mGPS_2 (*P* = 0.003).

**Fig. 3.**
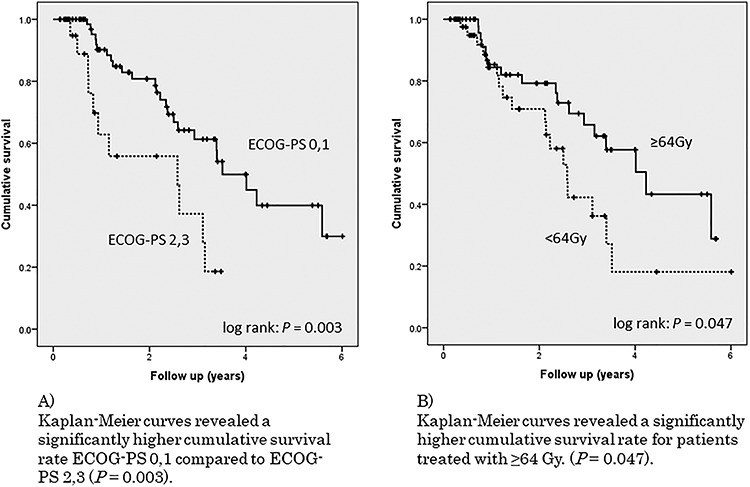
A) Kaplan–Meier curves revealed a significantly higher cumulative survival rate ECOG-PS 0,1 compared with ECOG-PS 2,3 (*P* = 0.003). B) Kaplan–Meier curves revealed a significantly higher cumulative survival rate for patients treated with ≥64 Gy. (*P* = 0.047).

Kaplan–Meier curves revealed a significantly higher cumulative survival rate for mGPS_0 compared with mGPS_1 and mGPS_2 (*P* = 0.003, Fig. 2). The univariate analysis of other factors, including good ECOG-PS (0 or 1) and higher-dose prescriptions (≥64 Gy), also exhibited significantly higher OS rates (Fig. 3A, 3B). There was no difference in OS rate with respect to age, sex, clinical stage, with or without TUR-Bt, with or without concurrent chemotherapy, or irradiation field. Multivariate analysis revealed that mGPS_0 (HR 0.37, *P* = 0.008) and good ECOG-PS (HR 0.39, *P* = 0.014) were independent good prognostic factors (Table 2). With respect to HR, we found that mGPS_0 had the greatest impact on the good prognosis of radiotherapy for MIBC.

## Discussion

Since CRP is one of the acute phase proteins derived from hepatocytes stimulated by circulating interleukin 6 (IL-6), CRP levels in cancer patients reflect the amount of IL-6 in the circulating blood produced by T-cells or macrophages. The chronic increase in CRP levels is due to the sustained increase of these inflammatory cell activities. The inflammatory cytokines also induce a reduction of serum albumin as CRP production is prioritized, and the kinetics promote exacerbated hypoalbuminemia when patients fail to intake required nutrition in disorders such as cachexia. In cancer patients, high mGPS suggests the existence of cachexia, independent of the clinical stage of cancer [14].

Multivariate analysis of this study showed that mGPS and ECOG-PS were prognostic factors for patients with MIBC after radiotherapy, and their respective HRs showed that mGPS exhibited a stronger impact on OS. It has been reported that mGPS is correlated with the outcome of surgery and chemotherapy for bladder cancer. Ferro et al. [11] revealed that in 1037 patients who underwent radical cystectomy plus pelvic lymphadenectomy, subjects with an mGPS equal to 2 had a significantly shorter median recurrence-free survival compared with subjects with an mGPS equal to 1 (16 vs. 19 months, HR 1.54, 95% confidence interval [CI] 1.31–1.81, *P* < 0.001) or with subjects with an mGPS equal to 0 (16 vs. 29 months, HR 2.38, 95% CI 1.86–3.05, *P* < 0.001). Hwang et al. [12] reviewed the OS rate of 67 patients who received systemic chemotherapy as first-line treatment for advanced bladder cancer and indicated that a systemic inflammatory response coupled with hypoalbuminemia (GPS_2) correlates with a shortened OS rate. As a postradiotherapeutic prognostic factor, Hannisdal et al. [15] reported in 1993 that the following five variables were significantly associated with shorter survival as prognostic factors in 202 bladder cancer patients treated with radiotherapy: T4 tumors, erythrocyte sedimentation rate > 30 mm/h, albumin < 35 g/l, lactate dehydrogenase > 400 U/I, and age > 75 years. This report predates the establishment of the concept of GPS by McMillan et al [4]. These reports corroborate our results and we are the first to document that mGPS is a good prognostic factor for MIBC during radiotherapy.

In addition, there are several reports that ECOG-PS is a prognostic factor in patients treated for bladder cancer. Tran E et al. [16] showed that younger age and good performance status were favorable prognostic factors for OS in elderly MIBC patients treated with radiotherapy. Weizer AZ et al. [17] revealed that Karnofsky performance status is an important predictor of OS in elderly patients with MIBC (75% were treated with cystectomy, and the others were treated with palliative treatment including bladder-sparing therapy).

On the other hand, no other background prognostic factor affects OS rates, such as TNM stage. Of the 98 MIBC patients we reviewed, 92% were elderly, and 51% of the patients had one or more comorbidities. In patients who are older and/or have comorbidities, it is easy to imagine that mGPS and ECOG-PS, which indicate the nutritional and physical status of the patient, affect OS rates more than tumor stage.

There were some limitations to this study. This retrospective cohort study included patients with various backgrounds and treatment methods, timing of blood sampling, follow-up period, and mGPS cutoff values.

Modified GPS may represent a novel biomarker that can predict survival in patients with MIBC after radiotherapy. Patient stratification by prognostic prediction may be useful for determining treatment strategies. Therefore, the development of new therapeutic strategies to improve this cachectic condition may be useful to improve the prognosis of elderly MIBC receiving radiotherapy.

## Presentation at a conference

ESTRO 37, April 20–24, 2018, Barcelona, Spain.
